# Dynamics of Bagaza, West Nile, and Usutu Viruses in Red-Legged Partridges, Portugal, 2018–2022 

**DOI:** 10.3201/eid3104.241293

**Published:** 2025-04

**Authors:** Catarina Fontoura-Gonçalves, Francisco Llorente, Elisa Pérez-Ramírez, Miguel Ángel Jiménez-Clavero, João Basso Costa, Gonçalo de Mello, David Gonçalves, Paulo Célio Alves, Ursula Höfle, João Queirós

**Affiliations:** Centro de Investigação em Biodiversidade e Recursos Genéticos (CIBIO), Porto, Portugal (C. Fontoura-Gonçalves, D. Gonçalves, P. Célio Alves, J. Queirós); Faculdade de Ciências, Porto (C. Fontoura-Gonçalves, D. Gonçalves, P. Célio Alves, J. Queirós); BIOPOLIS, Vairão, Portugal (C. Fontoura-Gonçalves, D. Gonçalves, P. Célio Alves, J. Queirós); IREC (CSIC-UCLM), Ciudad Real, Spain (C. Fontoura-Gonçalves, U. Höfle); Centro de Investigación en Sanidad Animal (CISA-INIA), Valdeolmos, Spain (F. Llorente, E. Pérez-Ramírez; M.Á. Jiménez-Clavero); CIBER of Epidemiology and Public Health, Madrid, Spain (M.Á. Jiménez-Clavero); Herdade de Vale de Perditos, Serpa, Portugal (J. Basso Costa, G. de Mello); EBM—Estação Biológica de Mértola, Mértola, Portugal (D. Gonçalves, P. Célio Alves, J. Queirós)

**Keywords:** *Orthoflavivirus*, viruses, zoonoses, wild birds, micro virus-neutralization test, sentinel species, One Health, Portugal, Bagaza virus, West Nile virus, Usutu virus

## Abstract

Long-term serologic surveillance of red-legged partridges suggests emergence of Bagaza virus in Portugal in 2021, associated with disease outbreaks in this species. Results also reveal sporadic circulation of Usutu virus and endemic circulation of West Nile virus, highlighting the role of red-legged partridges in the transmission and maintenance cycle and as sentinels of orthoflaviviruses.

Bagaza virus (BAGV), West Nile virus (WNV), and Usutu virus (USUV) are RNA viruses in the mosquitoborne cluster of the genus *Orthoflavivirus*, family *Flaviviridae.* Although BAGV recently emerged in Europe, WNV and USUV are both reemerging zoonotic viruses that can cause neurologic disease. First reported in Central African Republic in 1966 in *Culex* mosquitos (*1*), BAGV was detected in 2010 in southern Spain in an outbreak in red-legged partridges (*Alectoris rufa*) and pheasants (*Phasianus colchicus*) (*2*)*.* Since then, it has been detected sporadically in southern Spain, cocirculating with WNV and USUV (*2*). BAGV also caused disease outbreaks in Spain in 2019 (*3*) and in the south of Portugal in 2021 (*4*), affecting mainly partridges and 1 corn bunting (*Emberiza calandra*) (*4*). The Portugal outbreak raised the question of whether BAGV was introduced in Portugal for the first time in 2021 or whether it was already circulating with undetected deaths.

WNV and USUV cocirculate in many regions; incidence and distribution are increasing in Europe (*5*). WNV has been circulating in Spain since 2005; in 2020, it caused one of the largest human epidemics (*6*,*7*). In Portugal, the endemic circulation of WNV in wild birds remains unclear (*8*). USUV has been detected in Europe since 1996, affecting numerous avian species, and has been confirmed as zoonotic (*5*). No human or animal USUV cases have been reported in Portugal. The red-legged partridge, a game bird of substantial ecologic, economic, and social importance in Portugal and Spain (*9*), is highly susceptible to infection by orthoflaviviruses and is therefore a good model species for epidemiologic studies (*10*).

## The Study

**Figure 1:**
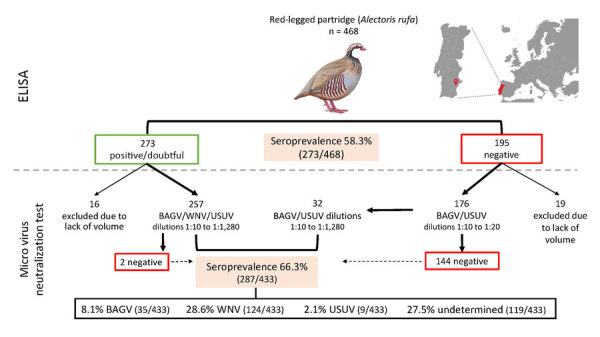
Diagnostic protocol and geographic location of sampling in study of dynamics of BAGV, WNV, and USUV in red-legged partridges, Portugal, 2018–2022. Diagnostic protocol was used to quantify the seroprevalence of WNV and WNV cross-reacting antibodies by ELISA and specific seroprevalence for BAGV, WNV, USUV, and undetermined orthoflaviviruses by virus neutralization test. Inset map shows location of Portugal in Europe (red shading) and location of red-legged partridge population sampled in this study (red location pin). BAGV, Bagaza virus; USUV, Usutu virus; WNV, West Nile virus.

Taking advantage of a long-standing surveillance program of a natural red-legged partridge population in southern Portugal (Vale de Perditos, Serpa; 37.82236N, −7.37953W), during October 2018–October 2022, we collected blood samples from 468 partridges and performed serologic analyses using a commercial competitive ELISA (Gold Standard Diagnostics, https://www.goldstandarddiagnostics.es) and micro virus neutralization test (VNT) against BAGV, WNV, and USUV (Figure 1). We evaluated individual (sex and age class) and temporal (season/year and month) drivers of orthoflavivirus exposure using general linear models (Appendix). 

This study was conducted as part of a surveillance program in a population of red-legged partridge, a common and highly managed game species in the Iberian Peninsula. The program included the routine capture, marking, and collection of biologic samples. All procedures were conducted by experienced researchers, ensuring the welfare of the animals and with legal permits granted by the Portuguese National Institute for the Conservation and Protection of Wildlife. The study was evaluated by the Animal Welfare Ethics and Review Body at BIOPOLIS-CIBIO (reference no. 2024–10).

**Figure 2:**
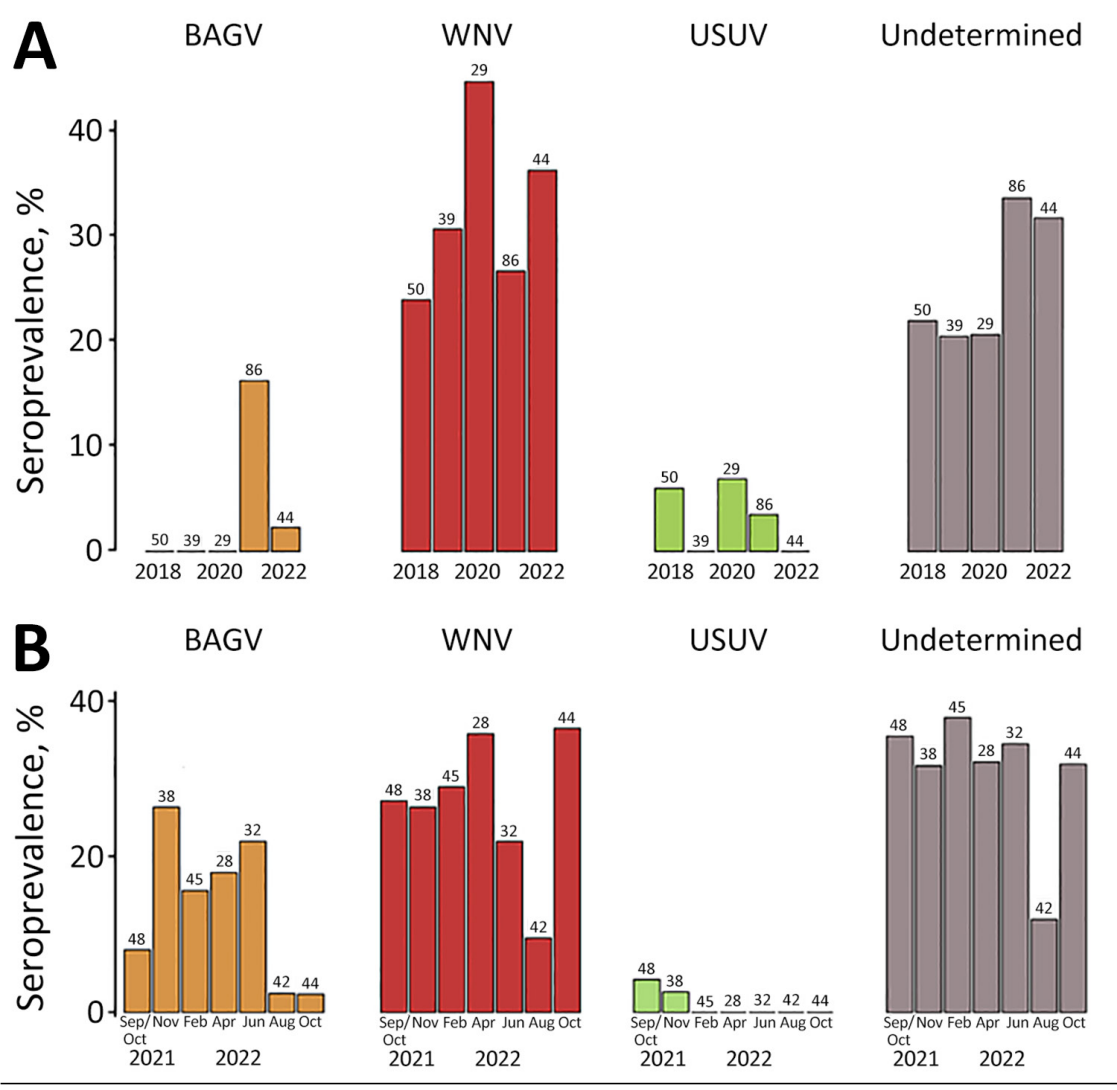
Seroprevalences obtained from micro virus neutralization test in study of dynamics of BAGV, WNV, and USUV in red-legged partridges, Portugal, 2018–2022. Results for BAGV, WNV, USUV, and undetermined orthoflaviviruses were determined in the autumn of all years (A) and at 2-month sampling intervals after the BAGV outbreak (B). The total number of samples analyzed is indicated in each column. BAGV, Bagaza virus; USSUV, Usutu virus; WNV, West Nile virus.

ELISA results revealed overall WNV or cross-reacting orthoflavivirus seroprevalence of 58.3% (273/468) (Figure 1; Appendix Table 1). VNT showed a 66.3% overall seroprevalence, identified as BAGV (8.1%, 35/433), WNV (28.6%, 124/433), USUV (2.1%, 9/433), or undetermined orthoflaviviruses (27.5%, 119/433) (Figure 1; Appendix Tables 2, 3). Seroprevalence varied between autumns of consecutive sampling years (Figure 2, panel A) and among the bimonthly sampling time points after the 2021 BAGV outbreak (Figure 2, panel B).

The dynamics of BAGV seroprevalence are consistent with its emergence in September 2021 (*4*). In subsequent months, seroprevalence remained stable until it decreased substantially in August and October of 2022 (Figure 2, panel B), when new juveniles captured and sampled for the first time (Appendix Figure 1) tested negative, suggesting that active circulation ceased or was very low during summer 2022.

The dynamics of WNV seroprevalence are consistent with endemic circulation in red-legged partridges. Although circulation was expected (*8*), the seroprevalences detected (ELISA 58.3%, VNT 28.6%) are higher than those reported previously in Portugal (9.8%) (*11*) and in Spain, where seroprevalences of 23% (*2*), 2.1%–17.5% (*7*), and 19.23% (*12*) have been reported. However, in contrast to previous studies, our WNV seroprevalence data correspond to only 1 highly susceptible species (*10*). The prevalence of WNV-specific antibodies was highest in October 2020 (44.8%), just after the human outbreak in southern Spain (*6*). Taken together, those data indicate that partridges might play a role in WNV transmission and maintenance and suggest the utility of this species as a sentinel for WNV circulation. Partridges are relatively abundant and easy to capture and, because they are a game species, sampling of hunted birds offers a cost-effective strategy for serologic and virological surveillance. Those aspects have been highlighted in other sentinel bird species, namely corvids (*13*). Corvids, especially magpies, are considered good WNV sentinels because of their broad distribution, abundance, sedentary behavior, and ease of capture, as well as their demonstrated high prevalences of WNV antibodies (*11*).

The dynamics of USUV seroprevalence are consistent with sporadic circulation in red-legged partridge, with specific antibodies being detected in autumn 2018, 2020, and 2021. Seroprevalences were lower than those detected in other studies conducted on game birds in Spain in 2011 (10%) (*2*). Of note, detection of USUV was confirmed in red-legged partridges in this region in November 2021 and November 2023 (J. Queirós et al., unpub. data, https://www.biorxiv.org/content/10.1101/2024.12.04.626753v1).

The results obtained for undetermined orthoflaviviruses could be explained by intrinsic limitations of laboratory methods, specifically those related to cross-reactions (no specific antibodies are detected even though the infection is caused by 1 of the 3 viruses), double infections by >1 orthoflavivirus (either successive or, less likely, simultaneous co-infections), or infection with an unknown orthoflavivirus (*14*)*.* The undetermined orthoflavivirus seroprevalences seem to follow the same tendency as for WNV, which seems to be modulated by the presence of USUV and BAGV (Figure 2). The increase of indistinguishable reactions to various orthoflaviviruses when USUV and BAGV first appeared suggests that those undetermined orthoflaviviruses could likely be explained by cross-reaction or infection (successive or simultaneous) by >1 virus (Appendix Figure 2). The fact that the mean seroneutralization titers (Appendix Figure 3) against different orthoflaviviruses exhibit a similar temporal pattern also suggests multiple exposures to different viruses.

Regarding the individual drivers of orthoflaviviruses exposure, the higher seroprevalence in adult partridges in autumn (Appendix Table 4) might be explained by their long life and consequently higher probability of exposure through mosquito bites. After the BAGV outbreak (Appendix Table 5), seroprevalence was generally higher in males (except for BAGV, USUV not included); however, those results must be interpreted with caution because sex differences in orthoflavivirus seroconversion are likely an interaction of complex behavioral, biologic, and ecologic features. Increasing sampling, both in geographic and temporal scales, would be crucial to understand possible drivers of exposure, especially because orthoflavivirus epidemiologic cycles appear to encompass longer time frames than in our study (*13*). Changes in sampling frequency are a limitation of our study; however, they reflect increased surveillance efforts after the 2021 BAGV outbreak. Restricted geographic range is another limitation; however, to maximize sampling effort, we focused only on 1 location, where sampling a considerable number of animals every 2 months was possible.

## Conclusions

Our long-term serologic surveillance of red-legged partridges suggests the emergence of BAGV in Portugal in September 2021, along with disease outbreaks (*4*). Our results also reveal sporadic USUV circulation and endemic WNV circulation in partridges in Portugal. The wild red-legged partridge is highly managed in the Iberian Peninsula and is susceptible to infection by a wide range of orthoflaviviruses, making this species optimal for monitoring and surveillance of those viruses, a feature that is not clearly established for other bird species. Continuous monitoring of partridges and other wild birds is key for understanding the epidemiologic dynamics of BAGV and other zoonotic viruses, such as WNV and USUV, in Portugal and Europe, including identifying risk factors associated with their emergence (BAGV and USUV) or persistence (WNV) in wildlife.

This article was published as a preprint at https://www.biorxiv.org/content/10.1101/2024.08.02.606292v1.

Appendix Additional information about dynamics of Bagaza, West Nile, and Usutu viruses in red-legged partridges, Portugal, 2018–2022.
