# Supplementary material for: Dynamics of Bagaza, West Nile, and Usutu Viruses in Red-Legged Partridges, Portugal, 2018–2022
Source: Emerg Infect Dis. 2025 Apr;31(4):824–8. doi: 10.3201/eid3104.241293 (PMC11950255; doi:10.3201/eid3104.241293)
Supplement: Appendix — Additional information about dynamics of Bagaza, West Nile, and Usutu viruses in red-legged partridges, Portugal, 2018–2022. [file 24-1293-Techapp-s1.pdf]

Article DOI: https://doi.org/10.3201/eid3104.241293

EID cannot ensure accessibility for supplementary materials supplied by authors. Readers who have difficulty accessing supplementary content should contact the authors for assistance.

Dynamics of Bagaza, West Nile, and Usutu Viruses in Red-Legged Partridges, Portugal, 2018–2022

Appendix

Material and methods

Study area, sampling, and data collection

This study was conducted in Vale de Perditos, a hunting estate in the Municipality of Serpa (37.82236N, −7.37953W), Southern Portugal, between October 2018 and October 2022 (Figure 1, main manuscript). During these 5 years, partridges were sampled at least once a year, after the reproductive season (October-December). In 2019, an additional sampling was performed in February. After the BAGV outbreak in 2021 (*1*), we increased the sampling effort and samples were collected approximately every 2 months (Table S1 indicates the sampling time points and number of samples per sampling event).

Samples were obtained either after the live capture of partridges or during necropsies of hunted/dead specimens. Live partridges were captured using baited walk-in traps. Each partridge was ringed and age and sex recorded. The age was determined by examining the primary feathers, while the sex was identified based on spur characteristics. Blood (<1% body mass) was collected using 1 ml syringes and stored in sterile Eppendorf tubes. Partridges were released at the capture site after sampling. In total, we collected 468 blood samples that were allowed to clot at room temperature and then maintained at 4°C for at least 2 h and a maximum of 24 h before centrifugation for 10 min at 12,000 rpm to separate serum and cellular fractions. Serum samples were maintained at −20°C until further analyses. Legal permissions to capture and mark the animals were provided annually by the Portuguese National Institute of Wildlife Conservation and Protection (ICNF).

Antibody detection assay

Initial screening for *Orthoflavivirus* antibodies was performed using a commercial competitive ELISA (Ingezim West Nile Compac, Ingenasa Spain), following the manufacturer´s instructions. Despite being designed for the detection of WNV antibodies, this diagnostic test has been shown to cross-react with antibodies directed against other orthoflaviviruses, like USUV and BAGV (*2*).

To assess the specific seroprevalence of each virus a micro virus neutralization test (VNT) against WNV, USUV and BAGV was performed, as it is considered the gold standard method for WNV serologic diagnosis (*3*). WNV lineage 1 strain Eg-101/NY99, USUV strain SAAR1776 and BAGV strain Spain/2010 were used for the VNT in Vero cell cultures as previously described (*2*). The VNTs were performed in the BSL-3 laboratory at Centro de Investigación en Sanidad Animal (CISA-INIA), CSIC. In the case of positive and doubtful ELISA samples, neutralizing antibody titers were determined in parallel for each serum sample against the three viruses using serial (2-fold) serum dilutions (1:10–1:1280/10240), while negative ELISA samples were analyzed in parallel only against USUV and BAGV using two serial dilutions (1:10–1:20), due to the higher specificity of the ELISA test to WNV when compared with USUV and BAGV (*2*). In this latter case, samples with no neutralizing immune response (<1:10) were considered negative for the three viruses, whereas all others were considered positive and again tested by VNT to USUV and BAGV in parallel, this time using serial dilutions 1:10–1:1280 (Figure 1, main manuscript).

Specific neutralizing antibody responses were based on the comparison of VNT titers obtained against the two/three flaviviruses: the neutralizing immune response observed was considered specific for a given virus when VNT titers were at least 4-fold higher than the titer obtained against the other viruses. When VNT titer differences did not reach this threshold, the result was considered inconclusive and the specificity for a given *Orthoflavivirus* could not be determined (“undetermined orthoflaviviruses”) (*2*). For 35 samples VNT could not be performed due to insufficient serum volume.

Individual and temporal drivers of orthoflavivirus exposure

To determine the effect of individual (sex and age class) and temporal (year and month) drivers of *Orthoflavivirus* exposure in red-legged partridges, generalized linear models (GLM) with binomial distribution and logit link function were constructed. As dependent variable, we used the individual serologic status, classified by the results of ELISA and VNT: WNV, BAGV, USUV, “undetermined orthoflaviviruses” and general VNT (all the

positives to VNT). Since the sampling effort was not equal in all years, two datasets were analyzed: i) considering just one sampling per year, in autumn; and ii) considering the samplings every 2 months since the BAGV outbreak in September 2021. Distinct GLMs were constructed for each of the previous datasets. All statistical analyses were performed using R software, version 4.3.2.

Appendix Table 1. *Orthoflavivirus* seroprevalence (%) calculated using a commercial competitive West-Nile virus ELISA test for the red-legged partridge sampled between 2018 and 2022

Year	2018		2019		2020	2021		2022					
Season	Autumn		Winter	Autumn	Autumn	Autumn		Winter	Spring	Summer		Autumn	
Month	Oct	Nov/Dec	Feb	Dec	Oct	Sep/Oct	Nov	Feb	Apr	Jun	Aug	Oct	Total
Seroprevalence (%)	28.6 (4/14)	40.9 (18/44)	62.5 (25/40)	55.0 (22/40)	72.5 (29/40)	66.7 (34/51)	70.0 (28/40)	73.3 (33/45)	64.3 (18/28)	62.9 (22/35)	16.3 (7/43)	68.8 (33/48)	58.3 (273/468)
	37.9 (22/58)					68.1 (62/91)				37.2 (29/78)			

Appendix Table 2. *Orthoflavivirus* seroprevalence (%) calculated using a micro virus neutralization test (VNT) for the red-legged partridge sampled between 2018 and 2022. The number of individuals analyzed (n) and the results for each virus (BAGV, WNV, USUV, and "undetermined flaviviruses") are shown.

Year	2018		2019		2020	2021		2022					
Season	Autumn		Winter	Autumn	Autumn	Autumn		Winter	Spring	Summer		Autumn	
Month	Oct	Nov/Dec	Feb	Dec	Oct	Set/Oct	Nov	Feb	Apr	Jun	Aug	Oct	Total
Excluded due to lack of volume (n)	3	5	2	1	11	3	2	0	0	3	1	4	35
Seroprevalence (%)													
BAGV	0 (0/11)	0 (0/39)	0 (0/38)	0 (0/39)	0 (0/29)	8.3 (4/48)	26.3 (10/38)	15.6 (7/45)	17.9 (5/28)	21.9 (7/32)	2.4 (1/42)	2.3 (1/44)	8.1 (35/433)
WNV	27.3 (3/11)	23.1 (9/39)	36.8 (14/38)	30.8 (12/39)	44.8 (13/29)	27.1 (13/48)	26.3 (10/38)	28.9 (13/45)	35.7 (10/28)	21.9 (7/32)	9.5 (4/42)	36.4 (16/44)	28.6 (124/433)
USUV	9.1 (1/11)	5.1 (2/39)	2.6 (1/38)	0 (0/39)	6.9 (2/29)	4.2 (2/48)	2.6 (1/38)	0 (0/45)	0 (0/28)	0 (0/32)	0 (0/42)	0 (0/44)	2.1 (9/433)
Undetermined orthoflaviviruses	9.1 (1/11)	25.6 (10/39)	23.7 (9/38)	20.5 (8/39)	20.7 (6/29)	35.4 (17/48)	31.6 (12/38)	37.8 (17/45)	32.1 (9/28)	34.4 (11/32)	11.9 (5/42)	31.8 (14/44)	27.5 (119/433)
Total	45.5 (5/11)	53.8 (21/39)	63.2 (24/38)	51.3 (20/39)	72.4 (21/29)	75.0 (36/48)	86.8 (33/38)	82.2 (37/45)	85.7 (24/28)	78.1 (25/32)	23.8 (10/42)	70.5 (31/44)	66.3 (287/433)
	52 (26/50)					80.2 (69/86)				47.3 (35/74)			

Appendix Table 3. Number of individuals classified as BAGV, WNV, USUV and “undetermined orthoflaviviruses” for each micro virus neutralisation test (VNT) titer category. Titers with “≥” means that no higher dilutions were performed since they were not necessary to differentiate the specific antibodies.

Titers	BAGV	WNV	USUV	Undetermined orthoflaviviruses	Total
1:10	0	2	0	13	15
1:20	0	0	1	10	11
1:40	0	3	4	10	17
1:80	0	9	1	10	20
1:160	1	22	2	21	46
1:320	12	32	0	26	70
1:640	15	23	1	13	52
1:1280	0	3	0	14	17
≥1:1280	7	21	0	0	28
1:2560	0	3	0	1	4
1:5120	0	2	0	0	2
≥1:5120	0	1	0	0	1
≥1:10240	0	3	0	1	4

Appendix Table 4. Results of the GLMs analyzing the relationship between overall *Orthoflavivirus* seroprevalences calculated by ELISA and VNT (General VNT) in the autumn dataset (n = 277). Models were also constructed for the seroprevalence obtained for each virus in the VNT analysis: BAGV, WNV, USUV and “undetermined orthoflaviviruses.” Significant results are considered when the *p-value* <0.05 and are highlighted with bold and *.

Coefficients	Estimate	Standard Error	z-value	Pr(>|z|)
**ELISA ~ Sex + Age +Year**				
Intercept	0.015	0.393	0.038	0.9700
Sex Male	0.385	0.283	1.360	0.1738
**Age Juvenile**	**-1.034**	**0.292**	**-3.538**	**0.0004***
Year 2019	0.125	0.470	0.265	0.7908
**Year 2020**	**1.222**	**0.543**	**2.252**	**0.0244***
**Year 2021**	**0.941**	**0.408**	**2.308**	**0.0210***
**Year 2022**	**0.927**	**0.468**	**1.983**	**0.0474***
**General VNT ~ Sex + Age +Year**				
Coefficients	Estimate	Standard Error	z-value	Pr(>|z|)
Intercept	0.597	0.402	1.484	0.1377
Sex Male	0.077	0.298	0.259	0.7960
**Age Juvenile**	**-0.962**	**0.308**	**-3.127**	**0.0018***
Year2019	−0.399	0.470	−0.849	0.3959
Year2020	0.771	0.537	1.437	0.1508
**Year2021**	**1.197**	**0.429**	**2.793**	**0.0052***
Year2022	0.691	0.471	1.466	0.1426
**BAGV ~ Sex + Age +Year**				
Coefficients	Estimate	Standard Error	z-value	Pr(>|z|)
Intercept	−21.340	2750.266	−0.008	0.9938
Sex Male	0.303	0.589	0.515	0.6064
Age Juvenile	1.076	0.584	1.843	0.0654
Year2019	0.274	3915.762	0.000	0.9999
Year2020	0.047	4243.377	0.000	1.0000
Year2021	19.024	2750.266	0.007	0.9945
Year2022	16.805	2750.266	0.006	0.9951
**WNV ~ Sex + Age +Year**				
Coefficients	Estimate	Standard Error	z-value	Pr(>|z|)
**Intercept**	**-0.855**	**0.421**	**-2.033**	**0.0421***
Sex Male	0.202	0.290	0.698	0.4854
Age Juvenile	−0.572	0.307	−1.863	0.0625
Year 2019	0.031	0.504	0.061	0.9512
Year 2020	0.760	0.520	1.460	0.1442
Year 2021	−0.070	0.434	−0.163	0.8705
Year 2022	0.419	0.478	0.877	0.8705
**USUV ~ Sex + Age +Year**				
Coefficients	Estimate	Standard Error	z-value	Pr(>|z|)
**Intercept**	**-2.372**	**0.895**	**-2.652**	**0.0080***
Sex Male	−0.200	0.794	−0.252	0.8011
Age Juvenile	−1.520	1.100	−1.381	0.1672
Year 2019	−17.882	2795.815	−0.006	0.9949
Year 2020	0.304	1.042	0.292	0.7702
Year 2021	−0.465	0.942	−0.493	0.6219
Year 2022	−17.622	2601.053	−0.007	0.9946
**Undetermined orthoflaviviruses ~ Sex + Age +Year**				
Coefficients	Estimate	Standard Error	z-value	Pr(>|z|)
**Intercept**	**-0.902**	**0.439**	**-2.054**	**0.0400***
Sex Male	−0.195	0.297	−0.657	0.5113
Age Juvenile	−0.528	0.318	−1.662	0.0965
Year 2019	−0.247	0.556	−0.444	0.6574

Coefficients	Estimate	Standard Error	z-value	Pr(>|z|)
Year 2020	−0.129	0.598	−0.215	0.8296
Year 2021	0.529	0.445	1.188	0.2347
Year 2022	0.461	0.502	0.918	0.3587

Appendix Table 5. Results of the GLMs analyzing the relationship between overall *Orthoflavivirus* seroprevalences calculated by ELISA and VNT (General VNT) in the two-month dataset after the BAGV outbreak in September 2021 (n = 199). Models were also constructed for the seroprevalence obtained for each virus in the VNT analysis: WNV, BAGV and "undetermined orthoflaviviruses." USUV data are not present because no antibodies were detected in 2022. Significant results are considered when the *p-value* <0.05 and are highlighted with bold and *.

Coefficients	Estimate	Standard Error	z value	Pr(>|z|)
**ELISA ~ Sex + Age + Month**				
Intercept	−0.950	0.486	−1.956	0.0505
**Sex Male**	**0.781**	**0.372**	**2.100**	**0.0357***
Age Juvenile	−0.667	0.392	−1.700	0.0892
Month August	−1.394	0.761	−1.832	0.0669
Month February	−0.148	0.542	−0.273	0.7848
Month June	−0.482	0.619	−0.778	0.4367
Month October	0.216	0.533	0.404	0.6860
**General VNT ~ Sex + Age + Month**				
Coefficients	Estimate	Standard Error	z value	Pr(>|z|)
Intercept	−0.950	0.486	−1.956	0.0505
**Sex Male**	**0.781**	**0.372**	**2.100**	**0.0357***
Age Juvenile	−0.667	0.392	−1.700	0.0892
Month August	−1.394	0.761	−1.832	0.0669
Month February	−0.148	0.542	−0.273	0.7848
Month June	−0.482	0.619	−0.778	0.4367
Month October	0.216	0.533	0.404	0.6860
**BAGV ~ Sex + Age + Month**				
Coefficients	Estimate	Standard Error	z value	Pr(>|z|)
**Intercept**	**-1.654**	**0.602**	**-2.748**	**0.0060***
Sex Male	−0.034	0.505	−0.068	0.9460
Age Juvenile	0.568	0.533	1.065	0.2867
**Month August**	**-2.397**	**1.162**	**-2.063**	**0.0391***
Month February	−0.299	0.655	−0.457	0.6478
Month June	0.350	0.690	0.507	0.6123
**Month October**	**-2.372**	**1.134**	**-2.093**	**0.0364***
**WNV ~ Sex + Age + Month**				
Coefficients	Estimate	Standard Error	z value	Pr(>|z|)
Intercept	−0.950	0.486	−1.956	0.0505
**Sex Male**	**0.781**	**0.372**	**2.100**	**0.0357***
Age Juvenile	−0.667	0.392	−1.700	0.0892
Month August	−1.394	0.761	−1.832	0.0669
Month February	−0.148	0.542	−0.273	0.7848
Month June	−0.482	0.619	−0.778	0.4367
Month October	0.216	0.533	0.404	0.6860
**Undetermined orthoflaviviruses ~ Sex + Age + Month**				
Coefficients	Estimate	Standard Error	z value	Pr(>|z|)
Intercept	−0.950	0.486	−1.956	0.0505
**Sex Male**	**0.781**	**0.372**	**2.100**	**0.0357***
Age juvenile	−0.667	0.392	−1.700	0.0892
Month August	−1.394	0.761	−1.832	0.0669
Month February	−0.148	0.542	−0.273	0.7848
Month June	−0.482	0.619	−0.778	0.4367
Month October	0.216	0.533	0.404	0.6860

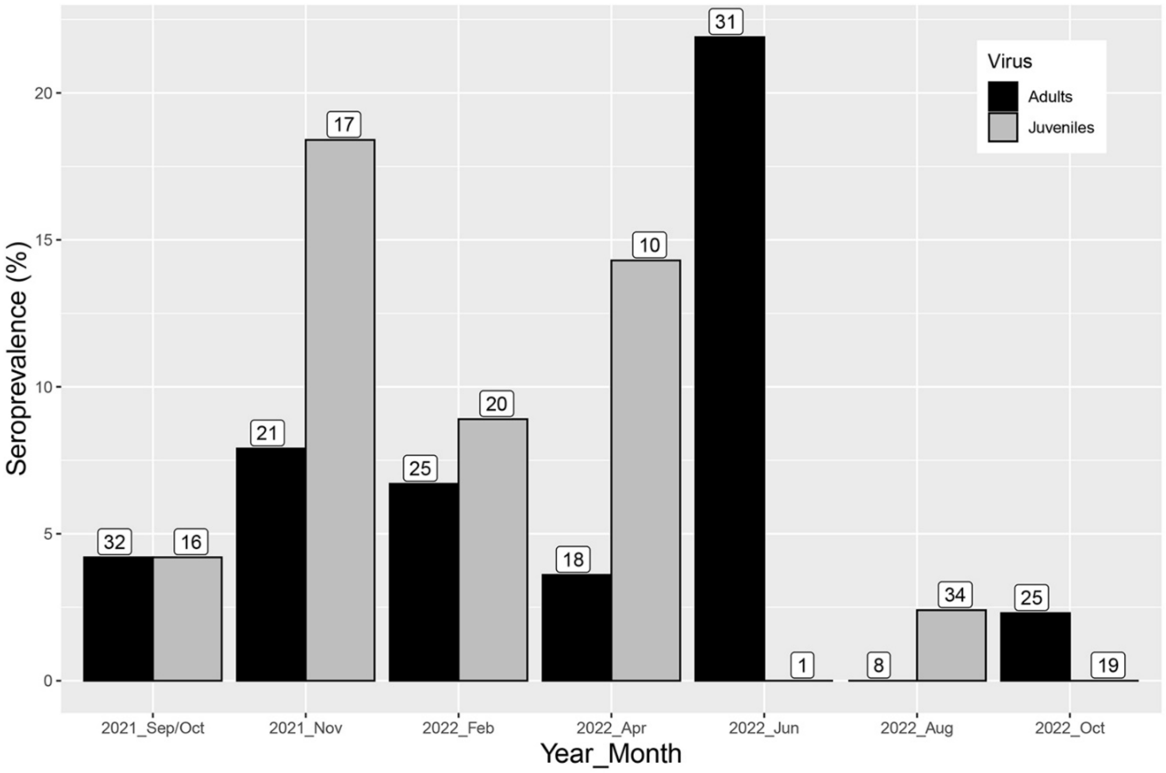

Appendix Figure 1. BAGV seroprevalence obtained by VNT in adults and juveniles after the BAGV outbreak in September 2021. Individuals born in 2021 are considered juveniles until spring 2022 (April). The number of samples analyzed is indicated in each column.

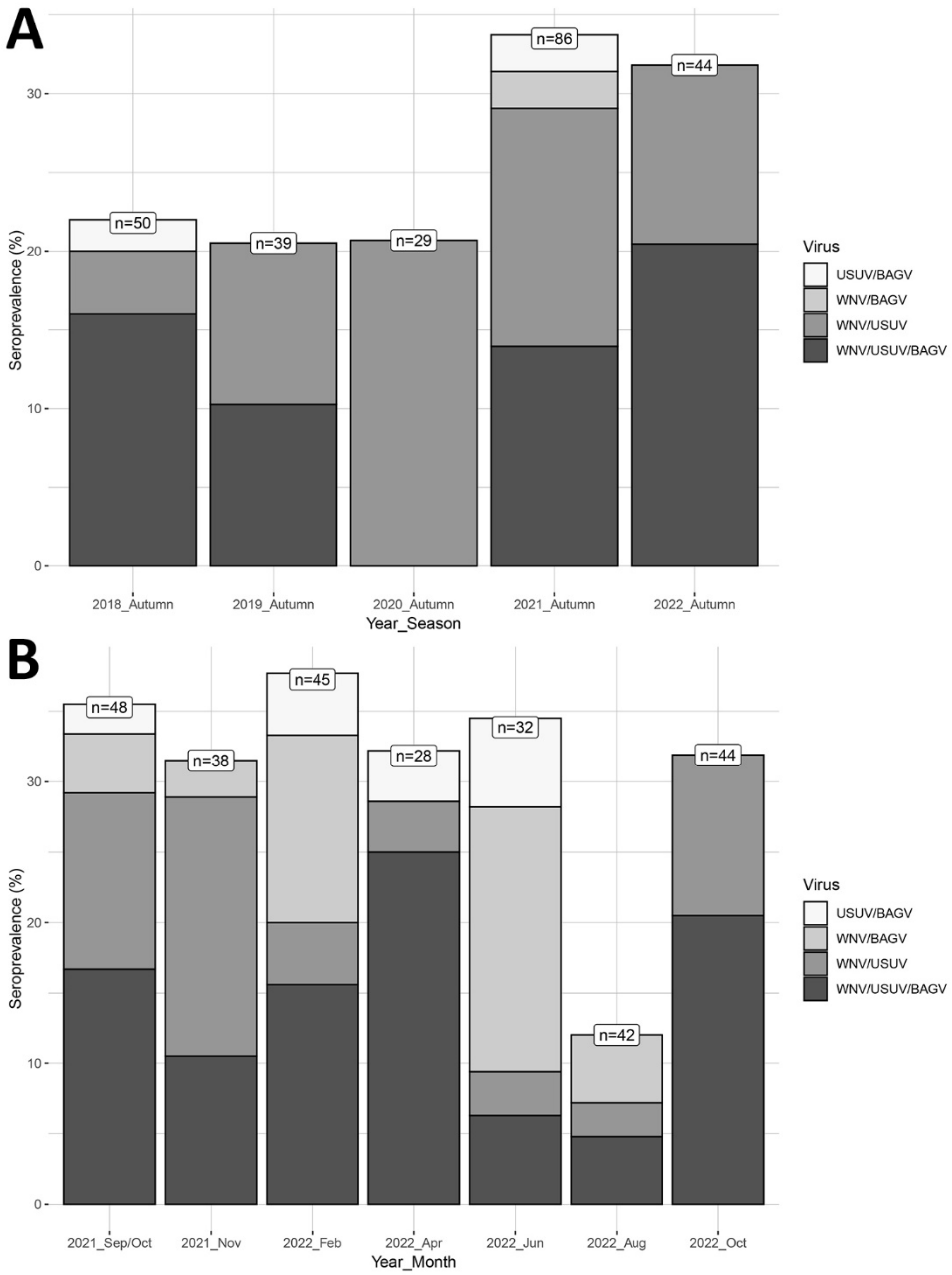

Appendix Figure 2. Seroprevalences of the different combinations of virus to which the sera considered “undetermined orthoflaviviruses” reacted to in the micro virus neutralization test (VNT): a) Throughout the autumn of all years; b) at 2-month sampling intervals after BAGV outbreak. On top of each column is the total number of samples analyzed.

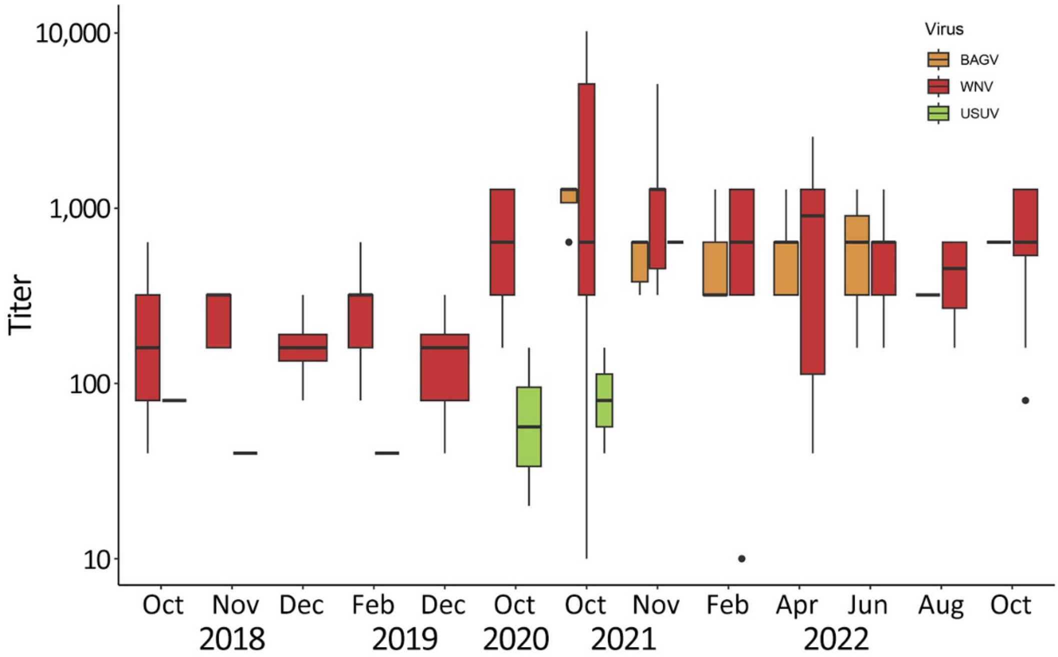

Appendix Figure 3. Distribution of the VNT titers to BAGV, WNV and USUV throughout all the sampling seasons. Titers are represented in Log 10 scale. Horizontal dark lines represent the median titer, the boxes represent the interquartile range (25th percentile – 75th percentile), the vertical lines (whiskers) the minimum and maximum values and the dots represent the outliers.

References

1. Queirós J, Barros SC, Sánchez-Cano A, Henriques AM, Fagulha T, Dos Santos FA, et al. Bagaza virus in wild birds, Portugal, 2021. Emerg Infect Dis. 2022;28:1504–6. PubMed https://doi.org/10.3201/eid2807.212408

2. Llorente F, García-Irazábal A, Pérez-Ramírez E, Cano-Gómez C, Sarasa M, Vázquez A, et al. Influence of flavivirus co-circulation in serological diagnostics and surveillance: a model of study using West Nile, Usutu and Bagaza viruses. Transbound Emerg Dis. 2019;66:2100–6. PubMed https://doi.org/10.1111/tbed.13262

3. OIE. West Nile fever. In: OIE Terrestrial Manual. 2008. p. 377–385.
